# Infection-Associated Pediatric Acute-Onset Neuropsychiatric Syndrome: A Review of Immunological Mechanisms, Clinical Phenotypes, and Therapeutic Strategies

**DOI:** 10.3390/idr18040069

**Published:** 2026-07-07

**Authors:** Enoch Chi Ngai Lim, Nga Chong Lisa Cheng, Chi Eung Danforn Lim

**Affiliations:** 1 Translational Research Department, Specialist Medical Services Group, Earlwood, NSW 2206, Australia; 2 National Institute of Complementary Medicine Health Research Institute, Western Sydney University, Westmead, NSW 2145, Australia; 3 Data Science Institute, University of Technology Sydney, Ultimo, NSW 2007, Australia

**Keywords:** PANS, PANDAS, infection, neuroinflammation, antineuronal antibodies, dopamine receptor antibodies, blood–brain barrier, immunomodulation

## Abstract

Background/Objectives: Pediatric acute-onset neuropsychiatric syndrome (PANS) describes the rapid onset of obsessive–compulsive symptoms or severe food restriction, accompanied by neuropsychiatric or somatic features that are not better explained by another disorder. PANDAS (Pediatric Autoimmune Neuropsychiatric Disorders Associated with Streptococcal infections) is a related, more narrowly defined construct in which symptoms are temporally associated with group A *Streptococcus* infection. This review examines clinical symptoms, infectious associations, proposed immune mechanisms, biomarker limitations, and treatment strategies for infection-associated PANS/PANDAS. Methods: A structured narrative search of PubMed/MEDLINE, Embase, the Cochrane Library, Google Scholar/publisher-indexed literature, ClinicalTrials.gov, and reference lists was performed up to 16 June 2026. Original cohorts, case series, systematic reviews, narrative reviews, consensus guidance, mechanistic studies, and registered prospective studies were prioritized. The review was not designed as a PRISMA-ScR scoping review; however, the methods were expanded to improve transparency and align with SANRA principles. Results: Group A *Streptococcus* remains the best characterized infectious association, although prospective studies have not uniformly demonstrated a consistent temporal relationship between streptococcal infection and neuropsychiatric exacerbations. Parent-reported surveys and case-based literature also describe temporal associations with *Mycoplasma pneumoniae*, influenza-like illnesses, upper respiratory infections, *Borrelia burgdorferi*, Epstein–Barr virus, and SARS-CoV-2. Proposed mechanisms include molecular mimicry, anti-D1R and anti-D2R antibodies, other antineuronal antibodies, calcium/calmodulin-dependent protein kinase II signaling, blood–brain barrier vulnerability, cytokine and Th17 effects, neuroinflammatory amplification, basal ganglia/CSTC circuit dysfunction, and gut–oral–brain immune interactions. None currently provides a definitive diagnostic biomarker. Conclusions: Infection-associated PANS is best approached as a clinically defined, heterogeneous neuroimmune presentation that requires rigorous differential diagnosis, multidisciplinary care, cautious treatment escalation, prospective biomarker validation, and large, multicenter treatment trials.

## 1. Introduction

The abrupt onset of obsessive–compulsive disorder (OCD), severely restricted food intake, tics, anxiety, regression, irritability, urinary symptoms, or cognitive deterioration in a previously stable child is clinically distinct from the gradual evolution typical of many primary psychiatric disorders. The PANDAS construct was introduced after Swedo and colleagues described 50 children with episodic OCD or tic disorders associated with group A streptococcal (GAS) infection [1]. The later PANS criteria broadened the phenotype beyond streptococcal disease by requiring abrupt onset of OCD or severely restricted food intake plus at least two additional neuropsychiatric symptom domains, while also requiring exclusion of better established neurological, medical, or psychiatric explanations [2]. This shift from a pathogen-specific label to a syndrome-based label increased clinical inclusiveness but also increased the need for careful diagnostic discipline.

The broadened definition is useful because some children present with a PANDAS-like acute neuropsychiatric syndrome without a clear GAS exposure. At the same time, PANS is not confirmed by a single laboratory test, imaging result, antibody panel, or treatment response. The evidence base includes parent-report surveys, expert consensus papers, small cohorts, case series, systematic reviews, clinical guidance, and a limited number of controlled intervention studies [3,4,5,6,7]. This mixed evidence base has generated controversy: some patients appear to have abrupt, relapsing–remitting neuropsychiatric disease that plausibly follows infection or inflammation, whereas other children may have primary OCD, tic disorders, eating disorders, or other medical conditions whose timing overlaps with common childhood infections. A cautious review must therefore acknowledge both clinical need and evidentiary uncertainty.

The infection-associated subgroup is particularly important because it sits at the boundary of pediatrics, psychiatry, neurology, infectious diseases, rheumatology, and immunology. In practice, families often seek help after a sudden change in behavior that appears temporally linked to sore throat, fever, respiratory infection, or another inflammatory exposure. Clinicians must decide whether to test for treatable infection, how to interpret positive or negative results, when to manage symptoms as primary psychiatric illness, and when to consider a broader neuroimmune evaluation. Over-attribution to infection can expose children to unnecessary antibiotics, procedures, and immunotherapies; under-recognition can delay treatment of infection, autoimmune encephalitis, Sydenham chorea, or severe psychiatric risk. The central clinical challenge is therefore not simply whether infection can trigger neuropsychiatric deterioration, but how to identify the subset of children in whom that model is most plausible.

This review focuses on infection-associated PANS/PANDAS and synthesizes four practical questions: which infectious triggers have been reported, what immune and neurobiological mechanisms have been proposed, how the phenotype should be evaluated, and how treatment should be selected in the setting of low-certainty evidence. The goal is not to present PANS as a single-pathway disease. Rather, the available literature supports a heterogeneous syndrome in which multiple infectious and inflammatory exposures may converge on vulnerable cortico–striato–thalamo–cortical (CSTC) circuits, producing abrupt changes in compulsivity, anxiety, motor symptoms, eating behavior, sleep, cognition, and autonomic or urinary function.

The contribution of this manuscript, relative to recent reviews and systematic reviews, is its targeted infectious-disease framing: it integrates the strength of microbial evidence, COVID-19-era literature, immunological mechanisms, antibody-assay limitations, neuroimaging, microbiome hypotheses, treatment risks, and practical diagnostic/management pathways into a single cautious clinical framework. The emphasis is on distinguishing documented infection from non-specific temporality, hypothesis-generating findings from established practice, and low-risk care from higher-risk antimicrobial or immunomodulatory escalation.

## 2. Methodology

This article is a structured narrative review. Peer-reviewed studies were identified from PubMed/MEDLINE, Embase, the Cochrane Library, Google Scholar and publisher-indexed literature up to 16 June 2026 using combinations of the terms PANS, PANDAS, pediatric acute-onset neuropsychiatric syndrome, streptococcal infection, *Streptococcus pyogenes*, *Mycoplasma pneumoniae*, SARS-CoV-2, COVID-19, Epstein–Barr virus, *Borrelia burgdorferi*, antineuronal antibody, dopamine receptor, D1R, D2R, Cunningham Panel, CaMKII, blood–brain barrier, basal ganglia, neuroimaging, microbiome, gut–brain axis, antibiotics, intravenous immunoglobulin, plasma exchange, and immunomodulatory treatment.

The inclusion approach prioritized English-language human pediatric and adolescent literature addressing abrupt-onset OCD, severe food restriction, tics, or acute neuropsychiatric deterioration in relation to infection, immune mechanisms, biomarkers, diagnostic evaluation, or treatment. Original cohorts, case–control studies, case series, mechanistic human studies, selected translational animal studies, systematic reviews, recent narrative reviews, consensus guidelines, and ClinicalTrials.gov records for active or recent prospective studies were included. Case reports were used only where they provided trigger-specific or mechanistic information not available from larger studies. Exclusion criteria were: non-pediatric focus without mechanistic relevance, gradual-onset psychiatric disorders without acute PANS/PANDAS relevance, commentary without clinically useful data, non-English reports without accessible English abstracts, and preprints not followed by peer-reviewed publication. Reference lists of key papers and reviews were manually searched; grey literature was not used except for ClinicalTrials.gov trial-status information.

**Figure 1:**
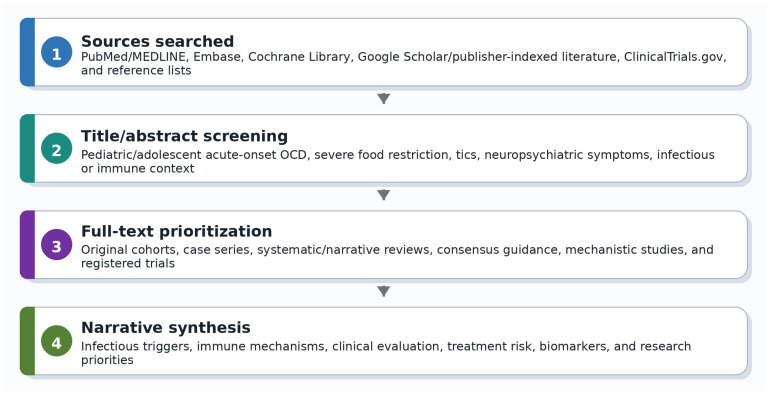
PRISMA-style transparency map for the narrative literature selection process. The figure illustrates source types, screening logic, full-text prioritization, and synthesis domains without implying a formal PRISMA-ScR count diagram. Abbreviations: MEDLINE, Medical Literature Analysis and Retrieval System Online; OCD, obsessive–compulsive disorder.

Records were screened by title and abstract, followed by full-text review of potentially relevant sources. Inclusion disagreements were resolved by consensus among the authors. Because this was not a registered systematic or scoping review, no meta-analysis was performed, and PRISMA-ScR was not used as a formal reporting framework. Instead, transparency was strengthened using SANRA domains for narrative reviews, and the literature-selection logic is summarized in Figure 1—a PRISMA-style transparency map rather than a quantitative PRISMA flow diagram [8,9]. This distinction is important because formal database record counts were not used to make pooled estimates or evidence grades.

The review emphasizes conservative interpretation of causality. Temporal association, even when clinically compelling, was not treated as proof that a pathogen caused the neuropsychiatric syndrome. Greater weight was given to reports with clearly described symptom acuity, standardized PANS/PANDAS criteria, documented infection or exposure, differential diagnosis, comparison groups, blinded or prospective design, and explicit treatment outcomes. Systematic reviews were used to frame the certainty of therapeutic evidence, while consensus guidance and clinical reports were used to describe current practice where trial data are limited [4,5,6].

## 3. Infectious Triggers and Strength of Evidence

Evidence for infectious triggers varies substantially by pathogen. The strongest evidence exists when a trigger is repeatedly reported, biologically plausible, temporally close to symptom onset, and supported by objective testing rather than retrospective recall alone. For PANS/PANDAS, these conditions are best met by GAS, most commonly *Streptococcus pyogenes*, although even GAS-associated presentations remain uncertain. Other pathogens, including respiratory viruses, *Mycoplasma pneumoniae*, Epstein–Barr virus (EBV), *Borrelia burgdorferi*, and SARS-CoV-2, have been reported to be associated with acute-onset neuropsychiatric symptoms, but the evidence is often case-based, retrospective, or complicated by non-specific serology. This difference in evidentiary strength should guide both clinical testing and the language used with families.

GAS has the strongest historical and mechanistic association with abrupt neuropsychiatric deterioration. In the original PANDAS cohort, temporal association with streptococcal infection was a defining feature rather than an independent estimate of incidence [1]. In a later survey of 698 families, infection was reported as the primary inciting factor in 65% of cases, and GAS was reported to be associated with the initial episode in 54% [10]. These data support clinical vigilance for GAS in children with abrupt OCD, tics, eating restriction, or relapse after a sore throat or scarlet-fever-like illness. They do not, however, establish that GAS is responsible for most cases in the general population, because the survey was based on families already identifying with a PANS/PANDAS framework and pathogen testing was not standardized.

A balanced interpretation also requires attention to studies that did not show a consistent association. Prospective and longitudinal studies of children with tics and/or OCD found that many exacerbations were not temporally linked to newly acquired streptococcal infection, and one prospective blinded cohort reported that more than three-quarters of exacerbations were not associated with GAS acquisition [11,12,13]. A Danish nationwide study found associations between streptococcal throat infection and subsequent mental disorders, including OCD and tic disorders, but such registry data cannot by themselves establish the PANDAS mechanism in individual children [14]. These mixed findings support a tiered view: GAS is the best characterized and most clinically actionable infectious association, but it should not be treated as a universal explanation for acute OCD or tic exacerbations.

Several practical issues complicate GAS interpretation. A throat culture, rapid antigen detection test, or nucleic acid amplification test can identify current pharyngeal GAS, but a positive result may reflect carriage rather than a causal trigger, particularly in school-aged children. Antistreptolysin O and anti-DNase B titers can support recent exposure when interpreted as paired or rising titers, but a single elevated value may reflect past infection and a normal value does not always exclude a recent infection. Prior antibiotic exposure, sampling delay, and local epidemiology can all alter test results. Consequently, infection testing should be integrated with the clinical timeline, physical examination, known exposures, and alternative diagnoses rather than used as a stand-alone confirmation of PANS.

Non-streptococcal triggers are less secure but clinically relevant. Frankovich and colleagues described five youth who met PANS criteria across different etiologies, including infectious and non-infectious contexts, illustrating that a similar phenotype can follow diverse inflammatory exposures [15]. Calaprice and colleagues reported recurrent flares associated with colds, sinusitis, *Mycoplasma pneumoniae*, influenza, EBV, and Lyme disease, but these associations were not prospectively adjudicated [10]. Upper respiratory infections are especially challenging because they are common in childhood and often coincide with sleep disruption, fever, school absence, family stress, and medication changes. In such situations, the infection may be a true immune trigger, a non-specific stressor, or simply a coincidental event in a child with a fluctuating psychiatric disorder.

Pathogen-specific interpretation also requires caution. *Mycoplasma pneumoniae* can be associated with neurological disease, but serology may remain positive after prior exposure and does not necessarily prove active infection. EBV reactivation may indicate immune stress rather than primary causality. Lyme disease should be considered only when exposure risk, geography, clinical manifestations, and testing strategy are appropriate; indiscriminate testing can lead to false-positive or clinically ambiguous results. Influenza and influenza-like illnesses may plausibly trigger systemic cytokine responses, but most published associations with PANS remain observational. These caveats do not exclude infectious contribution; rather, they reinforce the need to distinguish documented infection from broad inflammatory temporality.

SARS-CoV-2 has added a further post-infectious context. Reports describe new or worsened acute-onset neuropsychiatric symptoms after COVID-19, including case series with standardized symptom measures and emerging pediatric post-COVID-19 neuroimmune observations [16,17,18]. Because SARS-CoV-2 exposure has been widespread, temporal proximity alone is insufficient to define a syndrome. Nevertheless, the COVID-19 era has highlighted mechanisms relevant to PANS more broadly, including post-infectious immune dysregulation, cytokine-mediated neuropsychiatric symptoms, autonomic complaints, fatigue, and viral reactivation. Prospective post-infection cohorts may therefore help clarify which acute-onset psychiatric presentations represent coincidental onset, stress-related deterioration, or an immune-mediated post-infectious phenotype.

**Figure 2:**
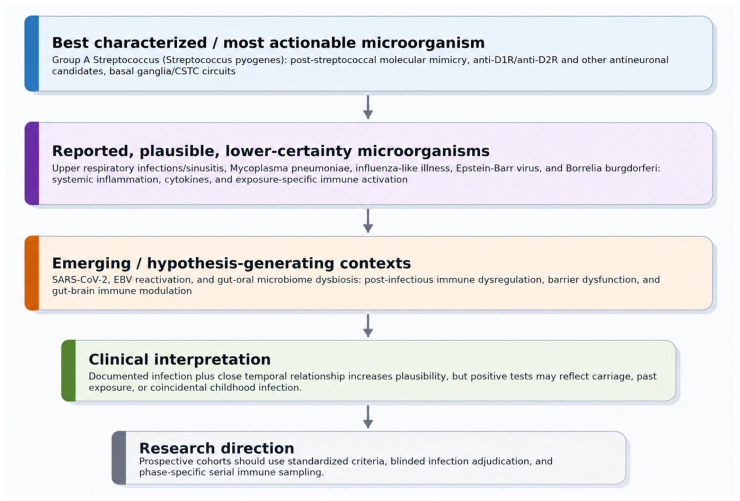
Graphical evidence map of reported infectious triggers. GAS/*Streptococcus pyogenes* remains the best characterized and most clinically actionable microbial association; other organisms are plausible but generally supported by lower-certainty evidence. Abbreviations: CSTC, cortico–striato–thalamo–cortical; D1R, dopamine receptor D1; D2R, dopamine receptor D2; GAS, group A *Streptococcus*; SARS-CoV-2, severe acute respiratory syndrome coronavirus 2.

Overall, infectious triggers should be discussed in tiers of certainty. GAS is the best characterized and most clinically actionable association. Other infections are plausible but less proven and should be evaluated only when the history, examination, epidemiology, and local clinical guidance support targeted testing. Table 1 summarizes reported triggers using cautious evidence language, and Figure 2 presents the same information graphically for infectious-disease readers.

## 4. Immunological and Neurobiological Mechanisms

Among the most developed mechanistic models is post-infectious immune cross-reactivity. Cunningham described how GAS antigen mimicry of host molecules can result in autoimmune sequelae [19]. In the PANDAS model, antibodies generated after infection are proposed to cross-react with neuronal targets, especially within basal ganglia-related circuits implicated in movement, habit learning, compulsivity, and emotional regulation. This model has intuitive clinical appeal because Sydenham chorea provides a precedent for post-streptococcal neuropsychiatric and movement symptoms. However, the PANS phenotype is broader than Sydenham chorea, and the presence of abrupt OCD or tics does not by itself prove a post-streptococcal autoimmune process.

The role of anti-dopamine receptor antibodies deserves explicit attention. In Sydenham chorea, anti-D1R and anti-D2R autoantibodies were higher than in controls and correlated with clinical neuropsychiatric symptoms, supporting dopamine receptor autoimmunity as one plausible bridge between infection and basal ganglia dysfunction [20]. More recently, Menendez and colleagues reported that dopamine receptor autoantibody signaling may differ between movement-predominant and neuropsychiatric post-infectious sequelae, with D1R and D2R titers/signaling proposed as mechanistic discriminators rather than validated stand-alone diagnostic tests [21]. These findings strengthen the biological rationale for dopamine receptor pathways in a subset of infection-associated cases, but they also require replication by independent laboratories.

Antineuronal antibody studies support immune involvement in at least some patients but have not produced a definitive diagnostic marker. Cox and colleagues found antineuronal antibodies and antibody-mediated neuronal signaling in a heterogeneous cohort of youth and young adults with tics and OCD, supporting the possibility that immune responses can influence neuronal pathways while also demonstrating that such markers are not specific to a single clinical label or trigger [22]. Other studies have reported less supportive findings; for example, Morris-Berry and colleagues found no single-point or longitudinal evidence that anti-tubulin or anti-D2R antibody levels distinguished PANDAS/Tourette groups from controls or tracked exacerbations [23]. Additional work on basal ganglia encephalitis and PANDAS has identified candidate autoantibody profiles, including D1R, D2R, lysoganglioside-GM1, tubulin, and CaMKII-related assays, but the clinical thresholds remain contested [24].

The translation of biomarkers into routine diagnosis has therefore been limited. Hesselmark and Bejerot evaluated the Cunningham Panel in suspected PANS/PANDAS and concluded that the relationship between panel results and symptomatology, as well as the panel’s diagnostic utility, remained uncertain [25]. Subsequent correspondence and evaluations illustrate the continuing controversy. Frye and Shimasaki argued that performance depends on assay conditions, patient selection, and interpretation [26], whereas Bejerot, Klang, and Hesselmark maintained concerns about reliability and commercial use [27]. A later retrospective study reported that changes in Cunningham Panel titers paralleled symptom changes in some patients, but this did not resolve concerns about selection bias, reproducibility, independent validation, and diagnostic specificity [28]. Clinically, antineuronal antibody results should be considered hypothesis-generating and should not be used alone to diagnose PANS, determine causality, or justify high-risk immunotherapy.

Blood–brain barrier (BBB) dysfunction offers a biologically plausible route by which peripheral immune responses could influence neural circuits. Pooni and colleagues reported cerebrospinal fluid abnormalities in children presenting for PANS evaluation, including findings related to protein and albumin quotients, but the study did not establish a cytokine signature applicable as a diagnostic test [29]. In a translational GAS model, repeated intranasal infection promoted CNS infiltration by streptococcal-specific Th17 cells, BBB breakdown, IgG deposition, microglial activation, and synaptic changes, all in the absence of viable bacteria in CNS tissue [30]. These data are mechanistically important, but animal findings do not prove the same cascade in every child with PANS.

Neuroinflammation may be best understood as an amplifier rather than a single cause. Infection-related cytokine release can alter sleep, appetite, pain sensitivity, threat perception, and cognitive efficiency. Systemic inflammation may also influence BBB permeability, allowing peripheral immune mediators to interact more readily with neural tissue. Microglial activation, even if transient, could amplify behavioral symptoms in circuits that regulate compulsive checking, avoidance, rage responses, motor inhibition, and interoceptive awareness. These processes are not unique to PANS; similar inflammatory and sickness-behavior pathways are observed in many infections. Their relevance to PANS lies in the abruptness, severity, clustering of symptoms, and relapsing–remitting course seen in some children.

The basal ganglia and CSTC pathways remain central to mechanistic thinking. OCD and tic disorders already implicate cortico–striatal loops, while PANS adds the question of why those loops might destabilize abruptly after infection or inflammation. A 2024 study of rigorously phenotyped PANS cases reported elevated IgG binding to striatal cholinergic interneurons during flares, with reduction during recovery, suggesting one potential cellular locus within basal ganglia circuitry [31]. This does not establish a diagnostic assay, but it supports the broader model in which immune mediators can affect striatal signaling relevant to compulsivity, tics, motor inhibition, and emotional regulation.

Neuroimaging findings also support basal ganglia interest while remaining insufficient for routine diagnosis. Early MRI work described enlarged basal ganglia volumes in children with OCD or tics associated with streptococcal infection [32]. A PET study using 11C-[R]-PK11195 reported increased binding in the caudate and lentiform nuclei in PANDAS and a more limited pattern in Tourette syndrome, which was interpreted as consistent with microglial activation [33]. Diffusion-weighted MRI studies in PANS have reported microstructural differences in deep gray matter and related regions [34]. These imaging studies are mechanistically useful, but they are small, heterogeneous, and not sufficiently specific to confirm PANS/PANDAS in an individual child. MRI, PET, EEG, and CSF studies are therefore best reserved for red flags, concerns for encephalitis, atypical course, or research protocols, rather than used as screening tests for all suspected PANS.

The gut–oral–brain axis has emerged as another hypothesis-generating field. Microbiome changes may influence immune tone, mucosal barrier function, inflammatory mediators, and neuroactive metabolites, and oral or gastrointestinal microbial patterns may interact with immune responses to infection. Recent reviews and Spalice-group work emphasize immune dysregulation and potential gut-barrier/endotoxemia signals, including a cross-sectional study in which children with PANDAS showed higher serum LPS, zonulin, NOX2-related oxidative stress, and isoprostanes than PANS and control groups [35,36,37]. These ideas are attractive because many families report gastrointestinal symptoms, dietary changes, antibiotic exposure, and fluctuating inflammation. However, controlled PANS-specific intervention data are lacking, and microbiome-directed strategies should not be presented as established disease-modifying treatment.

**Figure 3:**
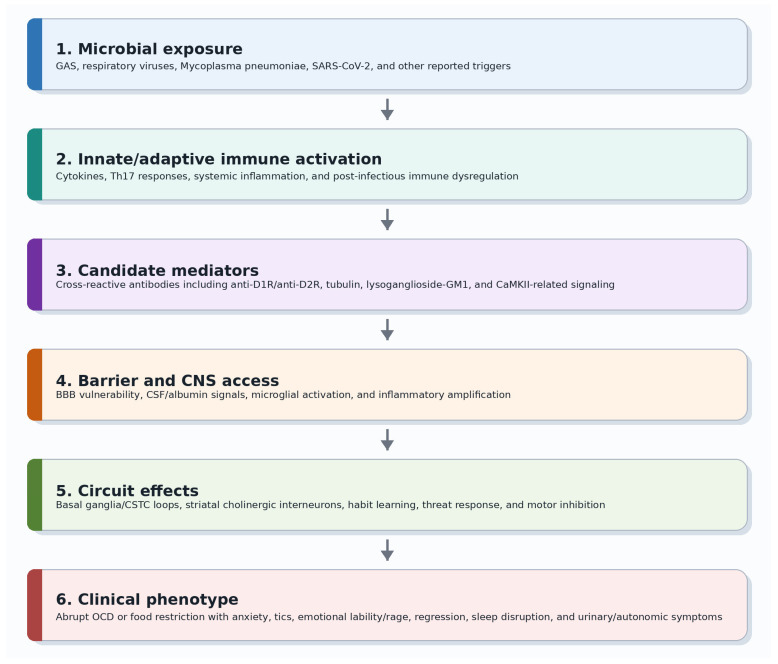
Proposed pluralistic pathophysiological cascade from infection to neuroinflammation and neuropsychiatric manifestations. Anti-D1R and anti-D2R antibody pathways are included as candidate mechanisms, not validated diagnostic markers. Abbreviations: BBB, blood–brain barrier; CaMKII, calcium/calmodulin-dependent protein kinase II; CNS, central nervous system; CSF, cerebrospinal fluid; CSTC, cortico–striato–thalamo–cortical; D1R, dopamine receptor D1; D2R, dopamine receptor D2; GAS, group A *Streptococcus*; GM1, monosialotetrahexosylganglioside; OCD, obsessive–compulsive disorder; SARS-CoV-2, severe acute respiratory syndrome coronavirus 2; Th17, T helper 17.

Taken together, the most defensible mechanistic model is pluralistic. GAS-associated PANDAS may involve molecular mimicry, dopamine receptor autoantibodies, and cross-reactive immune responses in some children, whereas other PANS presentations may reflect broader post-infectious immune dysregulation, inflammatory amplification, BBB vulnerability, microbiome–immune interactions, or interactions between infection and pre-existing neuropsychiatric risk. The absence of a definitive biomarker should not invalidate a carefully observed clinical syndrome, but it should prevent overconfident causal claims. Mechanistic uncertainty also supports a proportional approach to treatment: lower-risk psychiatric, behavioral, and targeted infectious interventions should be prioritized, while invasive immunomodulation should be reserved for selected cases after careful differential diagnosis. Figure 3 summarises this cascade. This cascade is proposed and pluralistic. It does not prove causality in an individual child and should not replace differential diagnosis or validated biomarkers.

## 5. Clinical Phenotype and Evaluation

PANS is defined by acuity. Parents often describe a clear inflection point, sometimes naming the day when a child suddenly developed contamination fears, reassurance seeking, checking, intrusive harm fears, refusal to eat, separation anxiety, rage, handwriting deterioration, urinary frequency, sleep disruption, or regression [2,10]. This acuity distinguishes PANS from many chronic developmental or psychiatric trajectories, but it does not remove the need for standard psychiatric and medical evaluation. Acute onset can occur in primary psychiatric disorders, autoimmune encephalitis, seizures, delirium, endocrine disease, medication reactions, and psychosocial crises. The diagnosis should therefore begin with a structured timeline rather than with an assumption that infection is causal.

A useful clinical history reconstructs the weeks before onset, the first days of symptoms, and the subsequent course. Clinicians should ask about sore throat, fever, cough, sinus symptoms, rash, gastrointestinal illness, known school or household exposures, tick exposure, COVID-19 infection, vaccination timing, antibiotic use, steroid exposure, sleep loss, trauma, new medications, substance exposure, and family history of autoimmune or neuropsychiatric disease. The symptom timeline should be paired with functional information: school attendance, handwriting or academic decline, food and fluid intake, weight change, urinary symptoms, sleep duration, aggression, suicidality, and caregiver capacity. This approach helps distinguish a single acute episode from a flare of a longer illness and helps determine urgency.

Physical and neurological examination should be severity-driven. Vital signs, hydration status, nutritional status, rash, pharyngitis, lymphadenopathy, joint findings, abnormal movements, choreiform signs, focal neurological deficits, altered mental status, catatonia, and psychosis can change the diagnostic pathway. Medical instability from food restriction, dehydration, suicidality, aggression, or severe insomnia requires immediate management regardless of whether PANS is ultimately confirmed. Red flags for autoimmune encephalitis, seizures, Sydenham chorea, central nervous system infection, metabolic disease, or systemic autoimmune disease should prompt specialist evaluation rather than routine outpatient management of PANS [2,6,38].

PANS/PANDAS and autoimmune encephalitis overlap in that both can present with abrupt psychiatric or behavioral change, but they differ in diagnostic anchors and urgency. PANS centers on abrupt OCD or severe food restriction plus additional acute neuropsychiatric domains and exclusion of better explanations. Autoimmune encephalitis is more likely when psychiatric symptoms are accompanied by altered level of consciousness, subacute memory loss, seizures, focal neurological findings, movement disorders beyond tics, CSF pleocytosis, MRI abnormalities, EEG abnormalities, or specific neuronal surface antibodies [6,38]. This distinction is clinically important: missing autoimmune encephalitis can delay urgent immunotherapy, whereas mislabeling PANS as encephalitis can expose children to unnecessary invasive testing and immunomodulation.

A focused infectious assessment is reasonable when the history or examination suggests a plausible trigger. Throat examination and GAS testing are appropriate when there are symptoms of pharyngitis, known exposure, scarlet-fever-like features, or a flare pattern strongly linked to streptococcal infection. Testing for *Mycoplasma pneumoniae*, EBV, SARS-CoV-2, influenza, or Lyme disease should be targeted to compatible symptoms, exposure risk, local epidemiology, and accepted pediatric guidance rather than ordered as a broad screening panel [2,6,39]. This targeted approach reduces false-positive results, minimizes unnecessary treatment, and preserves the clinical value of a positive test when it occurs in the right context.

Laboratory and biomarker testing should be interpreted conservatively. Basic studies may be indicated to assess inflammation, infection, nutritional compromise, thyroid disease, anemia, medication effects, or systemic illness, but normal inflammatory markers do not exclude PANS and abnormal markers do not prove it. Antineuronal antibody panels, CSF findings, EEG, MRI, PET, and other imaging may be considered in selected specialist evaluations, particularly when neurological red flags are present, but they are not stand-alone diagnostic tests [25,29,32,33,34]. A normal scan is common in functional psychiatric presentations and does not settle the question of immune involvement; an abnormal scan should prompt a broader neurological differential rather than automatic attribution to PANS.

The psychiatric assessment should be as rigorous as the medical workup. OCD symptoms should be characterized by obsessions, compulsions, avoidance patterns, insight, accommodation, and time burden. Food restriction should be evaluated for fear of contamination, choking, vomiting, body image concerns, sensory intolerance, nausea, or delusional beliefs, because each pathway has different safety implications. Mood lability, rage, anxiety, school refusal, tics, sleep disturbance, regression, and cognitive complaints should be documented with baseline comparisons and, where possible, standardized scales. This information provides a starting point for treatment and creates objective anchors for assessing improvement or relapse.

Differential diagnosis is not a competing exercise but a core part of PANS care. Clinicians must consider autoimmune encephalitis, Sydenham chorea, primary OCD, Tourette syndrome, eating disorders, delirium, seizures, medication effects, substance exposure, endocrine disease, and sleep disorders [2,6,38]. Some children will meet PANS criteria and also have a pre-existing neurodevelopmental or psychiatric vulnerability. Others may have infection-triggered worsening of an established disorder rather than a de novo syndrome. A formulation that acknowledges comorbidity is often more clinically useful than a binary decision between immune and psychiatric explanations.

Communication with families is crucial. Families frequently arrive after frightening behavioral changes and may have encountered polarized opinions about PANS. A balanced explanation validates the sudden deterioration, explains that infection and inflammation are being evaluated, and makes clear that psychiatric treatment does not dismiss immune mechanisms. It is also important to set expectations: improvement may require concurrent management of compulsions, sleep, school stress, infection, inflammation, and family accommodation. The practical goal is to identify treatable contributors, protect safety, reduce impairment, and avoid both therapeutic nihilism and unproven escalation.

Primary care clinicians can provide a structured first response before tertiary neuroimmune or psychiatric referral. The initial tasks are to confirm the abrupt timeline and last-known baseline, document core symptoms and functional impairment, assess immediate safety (food/fluid refusal, dehydration, weight loss, suicidality, aggression, psychosis, catatonia, severe insomnia, medication/substance exposure, and caregiver capacity), and perform a focused examination for pharyngitis, rash, abnormal movements, focal deficits, altered mental status, and systemic illness. When the child is medically or psychiatrically unstable, has encephalitic or focal neurological red flags, or cannot maintain hydration/nutrition, same-day emergency, neurology, infectious disease, or child psychiatry evaluation is warranted. If stable, primary care can initiate low-risk measures while referral is arranged: targeted GAS testing/treatment when indicated, avoidance of broad non-specific pathogen or antineuronal panels as screening tests, sleep and school supports, safety planning, family guidance to reduce accommodation, and baseline symptom tracking for OCD burden, food intake, weight, sleep, urinary symptoms, tics, rage episodes, and school attendance [2,6,39,40,41].

## 6. Management Principles

Treatment should address three parallel domains: psychiatric and functional stabilization, treatment or prevention of infection when documented or strongly suspected, and anti-inflammatory or immunomodulatory therapy for selected inflammatory presentations [39,40,41,42]. The relative emphasis varies by child and by phase of illness. A child with mild OCD symptoms after a resolved viral illness may primarily need psychoeducation, sleep restoration, school support, and exposure-based therapy. A child with abrupt food refusal, dehydration, psychosis, or suicidality requires urgent safety management. A child with repeated GAS-positive exacerbations may need coordinated pediatric and infectious disease review. Treatment planning should therefore begin with severity, risk, and diagnostic confidence rather than with a fixed protocol.

Psychiatric care is not secondary or optional. Consensus guidance recommends cognitive-behavioral therapy with exposure and response prevention (CBT/ERP), family-based support, school accommodations, sleep restoration, and careful pharmacotherapy when indicated [6,41]. In PANS, CBT/ERP may need modification because symptoms can be abrupt, intense, and accompanied by regression, rage, separation anxiety, or fluctuating capacity. Initial goals may include reducing family accommodation, restoring routines, improving sleep, and building tolerance for small exposures before more formal OCD work. School plans may need temporary flexibility around attendance, handwriting, urinary frequency, fatigue, and cognitive load while maintaining gradual reintegration.

Psychotropic medication can be useful but should be individualized. Selective serotonin reuptake inhibitors, sleep interventions, anxiolytic strategies, or medications for severe mood and behavioral symptoms may be considered using pediatric psychiatric principles, but families should be warned that activation, agitation, sedation, appetite changes, or worsening irritability can be difficult to distinguish from disease fluctuation [6,41]. Starting doses are often conservative, titration should be deliberate, and response should be measured against specific target symptoms. Medication should not replace behavioral treatment, safety planning, or investigation of medical red flags, but it can reduce suffering and improve the child’s capacity to participate in therapy.

Antimicrobial therapy should be targeted rather than indefinite. Consensus infection guidance supports treatment of documented GAS and other bacterial infections in accordance with accepted pediatric standards and discusses prophylaxis for carefully selected children with recurrent GAS-associated exacerbations [39]. This approach differs from treating every flare with antibiotics regardless of evidence. Systematic reviews and the AAP clinical report emphasize that routine long-term antibiotics for all PANS presentations are not established, and antimicrobial stewardship, adverse effects, allergy risk, gastrointestinal complications, Clostridioides difficile risk, microbiome disruption, and resistance must be weighed carefully [4,5,6]. When antibiotics are used, clinicians should document the indication, test results, expected duration, symptom targets, and plan for reassessment.

Anti-inflammatory treatment is sometimes used when symptoms suggest an inflammatory flare, but the evidence remains low certainty. Non-steroidal anti-inflammatory drugs and short courses of corticosteroids may be considered in some specialist settings after infection and safety concerns have been addressed, particularly when symptoms are moderate, time-limited, and accompanied by inflammatory features [5,42]. Risks include gastrointestinal, renal, metabolic, mood, sleep, and immunosuppressive effects. Corticosteroids may rapidly alter behavior in some children, either improving inflammatory symptoms or worsening agitation, insomnia, mania-like symptoms, or infection risk. Anti-inflammatory trials should therefore be explicit, time-limited, and monitored with predetermined outcome measures.

Intravenous immunoglobulin (IVIG) and therapeutic plasma exchange occupy the highest-risk end of the treatment spectrum. They may be considered for selected moderate-to-severe, relapsing, or refractory presentations after careful differential diagnosis and specialist review [42,43,44]. More recent clinical experience, including Pavone et al.’s 55-patient severe/extreme PANS/PANDAS IVIG cohort and rehabilitation review, supports continued study of IVIG in carefully selected severe cases but remains limited by non-randomized design, selection effects, and concurrent care [45]. These interventions are not diagnostic tests; improvement after treatment does not prove the original cause, and lack of response does not exclude immune involvement. Potential harms include headache, aseptic meningitis, infusion reactions, hemolysis, thrombosis risk, line complications, hypotension, bleeding, infection, cost, and access inequity. Systematic reviews emphasize that certainty about efficacy remains low and that adverse effects are more predictable than benefit in many clinical contexts [4,5].

The pediatric risk–benefit balance should be made explicit before escalation. Overdiagnosis can expose children to unnecessary antibiotics, repeated blood draws, lumbar puncture, imaging under sedation, surgical procedures, corticosteroids, IVIG, or plasma exchange, while also shifting attention away from urgent psychiatric treatment, eating-disorder care, sleep stabilization, or safeguarding needs. Conversely, under-recognition of severe neuroimmune disease can delay care. A proportional model therefore requires: documented indications, specialist input for high-risk therapies, objective baseline measures, adverse-effect monitoring, and stopping rules if benefit is absent or harms emerge.

Other proposed interventions should be framed carefully. Tonsillectomy or adenoidectomy should not be recommended solely as disease-modifying PANS therapy; standard ear, nose, and throat indications should guide surgical decisions, and systematic reviews have not established symptom-control benefits for PANS/PANDAS [4,5]. Microbiome-directed approaches, probiotics, dietary changes, and gut–oral–brain strategies remain investigational adjunctive concepts rather than established treatments [36,37]. They may be considered for general health or specific gastrointestinal indications, but they should not delay evidence-based psychiatric care, treatment of documented infection, or evaluation of serious neurological and medical conditions.

A practical management plan should include measurable outcomes. Families and clinicians can track OCD time burden, food intake, weight, school attendance, sleep duration, urinary symptoms, rage episodes, tics, anxiety, and caregiver accommodation. Structured parent-reported instruments such as the PANS-related Pediatric Treatment Evaluation Checklist (PTEC) may provide an additional longitudinal framework for flare and treatment monitoring, but they should supplement rather than replace clinician-rated severity, safety assessment, and functional outcomes [46]. Tracking reduces reliance on global impressions during a fluctuating illness and helps prevent treatment escalation based solely on fear. It also allows de-escalation when symptoms improve and highlights relapse patterns that may warrant renewed infection testing or psychiatric adjustment. Because PANS care often involves multiple clinicians, shared documentation of baseline severity, interventions, adverse effects, and response is essential.

The safest overall strategy is stepwise and multidisciplinary. Low-risk interventions that improve function should be implemented early, while higher-risk medical therapies should require stronger evidence of severity, impairment, inflammatory involvement, and exclusion of mimicking disorders. This does not mean delaying urgent care; it means matching treatment intensity to risk. Table 2 summarizes therapeutic approaches in PANS/PANDAS, emphasizing that the table is a clinical evidence summary rather than a rigid treatment protocol.

**Figure 4:**
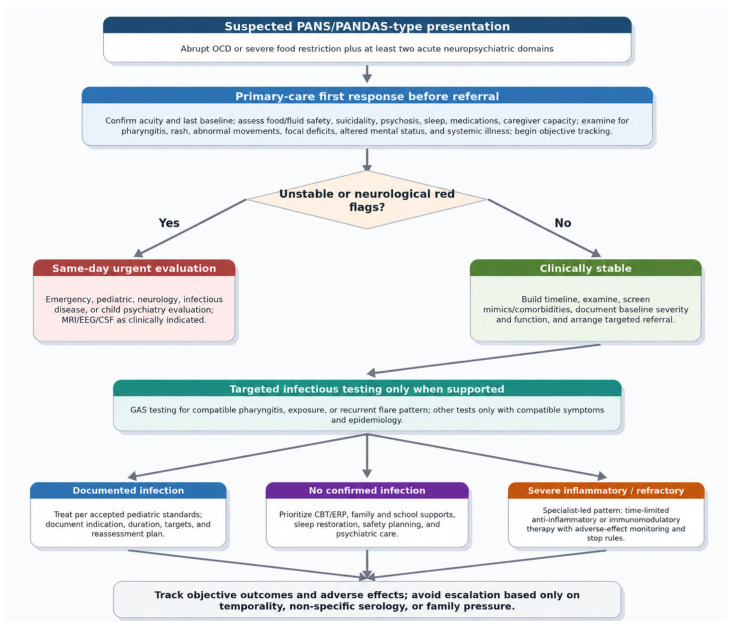
Graphical clinical algorithm for diagnosis and management. The algorithm emphasizes primary-care first response before referral, acuity, immediate safety, targeted infectious testing, differential diagnosis, first-line functional/psychiatric care, proportional treatment escalation, and adverse-effect monitoring. Abbreviations: CBT/ERP, cognitive-behavioural therapy with exposure and response prevention; CSF, cerebrospinal fluid; EEG, electroencephalogram; GAS, group A *Streptococcus*; MRI, magnetic resonance imaging; OCD, obsessive–compulsive disorder; PANDAS, Pediatric Autoimmune Neuropsychiatric Disorders Associated with Streptococcal infections; PANS, pediatric acute-onset neuropsychiatric syndrome.

To make clinical application explicit, Table 3 organizes bedside evaluation into staged decision points, Table 4 links the main mechanistic hypotheses to clinical and research interpretation, and Figure 4 provides a graphical diagnosis/management algorithm. These are intended to clarify reasoning rather than replace specialist assessment, local infectious disease guidance, or individualized risk assessment.

## 7. Limitations and Research Priorities

The PANS literature is constrained by heterogeneous case definitions, ascertainment bias, retrospective parent report, small samples, and variable infection testing. Some studies address PANDAS, others PANS, and many include mixed neuropsychiatric phenotypes. This heterogeneity makes causal inference difficult and partly explains why biomarkers and treatments have not translated cleanly into routine practice [3,4,5,6,7]. It also limits generalizability: a child treated in a tertiary neuroimmune clinic may differ substantially from a child with abrupt OCD seen in primary care, psychiatry, or emergency medicine.

A major limitation is the timing of infection assessment. In many reports, infectious testing occurs after symptoms have already evolved, after antibiotics have been started, or after families have reconstructed the timeline retrospectively. Common childhood infections create additional noise because exposure to GAS or respiratory viruses may be frequent even among children without PANS. Prospective designs are needed to determine whether infection rates, immune markers, and symptom trajectories differ from appropriate comparison groups with primary OCD, tic disorders, eating disorders, autoimmune encephalitis, Sydenham chorea, and healthy controls.

Future observational studies should use prospective inception cohorts with standardized PANS/PANDAS criteria, structured symptom instruments, blinded infection adjudication, serial immune profiling, and harmonized neuroimaging where feasible. They should capture not only symptom counts but also functional outcomes, including school participation, family accommodation, nutritional status, sleep, quality of life, and relapse frequency. A critical priority is to predefine standardized serial sampling protocols with common biospecimen types, processing procedures, assay platforms, and time windows. Sampling should be anchored to distinct clinical phases—acute onset or acute flare before major treatment changes when feasible, early/partial recovery, and stable remission—with comparator sampling in primary OCD, tic disorders, eating disorders, autoimmune encephalitis, Sydenham chorea, acute infection without neuropsychiatric symptoms, and healthy controls. Without phase-specific timing, potentially informative biomarkers may be missed, diluted, or misinterpreted.

### 7.1. Biomarker Discovery and Validation

Biomarker research should prioritize clinical utility rather than only group separation. A useful biomarker would help identify who has an infection-associated inflammatory phenotype, who is likely to relapse, who may benefit from immunomodulation, and who is at risk of adverse effects. Candidate approaches include transcriptomic, proteomic, cytokine, metabolomic, microbiome, neuroimaging, CSF, folate-metabolism, and autoantibody-based platforms. Current antineuronal antibody and CSF findings remain insufficient for routine diagnosis, but recent work on dopamine receptor signaling, striatal cholinergic interneuron binding, CSF characteristics, gut-barrier/endotoxemia markers, and folate receptor alpha autoantibodies (FRAAs) provides testable hypotheses for prospective validation [21,29,31,37,47].

Validation studies should predefine sample timing, comparator groups, assay platforms, thresholds, batch-effect handling, and clinical endpoints. They should include children with abrupt primary OCD, Tourette syndrome, eating disorders, Sydenham chorea, autoimmune encephalitis, systemic autoimmune disease, acute infection without neuropsychiatric symptoms, and healthy controls. Study protocols should specify serial collection at acute flare, early/partial recovery, and stable remission, with repeated symptom ratings collected at each biospecimen time point. Reporting should include sensitivity, specificity, likelihood ratios, reproducibility, phase-related change, and whether the biomarker changes management beyond what careful clinical assessment provides. Commercially available panels require particular caution because family demand and marketing can outpace independent validation [25,26,27,28].

### 7.2. Ongoing and Recent Studies

Treatment trials should separate acute infection treatment from anti-inflammatory and immunomodulatory strategies. A trial of antibiotics for documented GAS-associated exacerbations asks a different question from a trial of IVIG in severe relapsing PANS. Studies should stratify by severity, evidence of triggers, duration of illness, comorbidity, and prior treatment exposure. They should also include adverse-event capture, predefined response thresholds, blinded ratings where possible, and long-term follow-up. Because many families pursue multiple simultaneous treatments, trial designs must account for background psychiatric care, school support, and natural fluctuation.

Registered studies illustrate the field’s direction. A completed phase III ClinicalTrials.gov study compared Panzyga with placebo in PANS/PANDAS and posted results in January 2026; interpretation should await full peer-reviewed analysis and replication [48]. The PEDI PANDAS study is recruiting to test the feasibility of prospectively determining incidence, GAS contribution, and one-year natural history in children with recent-onset PANS/PANDAS [49]. A completed NIMH natural-history protocol collected clinical data and biospecimens to identify subgroups and environmental-pathogen roles [50], and a Stanford study is registered to investigate neurobiologic, immunologic, and rheumatologic markers in youth with PANS [51]. These studies reflect the need to move beyond retrospective association toward reproducible subgroups, validated outcomes, standardized serial sampling, and treatment decisions that improve function without unnecessary risk.

## 8. Conclusions

Infection-associated PANS is a clinically defined syndrome involving abrupt deterioration in neuropsychiatric function, most often characterized by sudden OCD symptoms or severe food restriction with additional behavioral, cognitive, motor, sleep, urinary, autonomic, or somatic features. GAS remains the best characterized infectious association, whereas *Mycoplasma pneumoniae*, EBV, influenza-like illnesses, Lyme disease, upper respiratory infections, and SARS-CoV-2 are reported with lower and more variable certainty. The current literature supports vigilance for treatable infection but does not support assuming that every acute psychiatric flare is infectious in origin.

Proposed mechanisms include molecular mimicry, anti-D1R and anti-D2R antibodies, other antineuronal antibody effects, CaMKII signaling, blood–brain barrier vulnerability, cytokine- and Th17-mediated amplification, microglial activation, basal ganglia/CSTC circuit dysfunction, striatal cholinergic interneuron involvement, and gut–oral–brain immune interactions. These models are biologically plausible and may overlap, but none currently provides a definitive diagnostic biomarker. PANS should therefore be evaluated through careful history, severity assessment, targeted infectious testing, rigorous differential diagnosis, and attention to psychiatric, neurological, and medical safety.

Treatment should be individualized and proportional. Psychiatric and behavioral care, family support, school accommodations, sleep restoration, and safety planning are foundational. Documented infections should be treated according to accepted pediatric standards, while prophylactic antibiotics, anti-inflammatory agents, IVIG, or plasma exchange should be reserved for selected situations in which severity, recurrence, evidence of inflammation, and specialist assessment justify the risk. Progress in the field will depend on large multicenter prospective studies, standardized diagnostic criteria, phase-specific serial biomarker sampling, validated biomarkers, and controlled treatment trials that distinguish infection-associated inflammatory disease from coincidental infection, primary psychiatric illness, and other neuroimmune disorders.

## Figures and Tables

**Table 1:** Infectious triggers reported in PANS/PANDAS literature and proposed pathogenic mechanisms.

Trigger	Evidence in Reviewed Literature	Mechanistic Interpretation	Evidence Caveats	Key Refs
Group A *Streptococcus* (GAS; *Streptococcus pyogenes*)	Original PANDAS cohort; 54% initial association in a 698-family survey; strongest historical and mechanistic literature.	Post-streptococcal molecular mimicry and cross-reactive antineuronal immune responses affecting basal ganglia/CSTC signaling; D1R/D2R and CaMKII pathways studied.	Best characterized trigger, but prospective incidence and diagnostic specificity remain uncertain; positive tests may reflect carriage or past exposure.	[1,10,11,12,13,14]
Upper respiratory infections, colds, sinusitis	Commonly reported with recurrent flares in survey data.	Non-specific systemic inflammation, immune priming, fever, sleep disruption, and possible BBB permeability changes; not pathogen specific.	Mostly retrospective parent report; pathogen confirmation often unavailable.	[10]
*Mycoplasma pneumoniae*	Reported in PANS case material and survey associations with initial episodes and recurrences.	Parainfectious immune activation is plausible; direct CNS involvement is recognized in Mycoplasma neurological disease but not proven as a PANS mechanism.	Evidence is case-based and observational; serology interpretation can be difficult.	[10,15]
Influenza or influenza-like illness	Reported as an infectious association with flares in survey data.	Cytokine-mediated systemic inflammation and neuroimmune amplification are biologically plausible.	The literature supports association, not causation; the survey does not show influenza as a dominant initial trigger.	[10]
*Borrelia burgdorferi*/Lyme disease	Reported in a minority of survey respondents; largely case-based in PANS literature.	Potential systemic or neuroinflammatory contribution; mechanism in PANS remains speculative.	Risk of over-attribution where Lyme testing is not clinically indicated or interpreted without exposure context.	[10]
Epstein–Barr virus (EBV)	Reported in survey recurrences and COVID-era reports describing EBV reactivation in some adolescents.	Immune activation or viral reactivation may contribute to inflammatory amplification.	Evidence is limited; EBV reactivation may be a marker of immune stress rather than the primary cause.	[10,16]
SARS-CoV-2	Case reports and pediatric case series describe acute-onset neuropsychiatric presentations following infection.	Post-infectious immune dysregulation, cytokine effects, autoantibody production, BBB disruption, and microglial activation have been proposed.	Temporal association is documented, but incidence, risk factors, and causality require prospective study.	[16,17,18]

Abbreviations: BBB, blood–brain barrier; CaMKII, calcium/calmodulin-dependent protein kinase II; CNS, central nervous system; CSTC, cortico–striato–thalamo–cortical; D1R, dopamine receptor D1; D2R, dopamine receptor D2; EBV, Epstein–Barr virus; GAS, group A Streptococcus; SARS-CoV-2, severe acute respiratory syndrome coronavirus 2. Note: Evidence categories are descriptive, not formal GRADE ratings.

**Table 2:** Summary of therapeutic approaches in PANS/PANDAS.

Therapeutic Approach	Clinical Role	Evidence Base	Cautions and Limitations	Key Refs
CBT/ERP, family and school supports, sleep and safety planning	First-line functional and symptom management; adapted to acuity, regression, rage, and fluctuating capacity.	Consensus psychiatric guidance plus established pediatric OCD treatment principles.	May require low-intensity starts during acute flares; monitor suicidality, eating restriction, aggression, and caregiver burden.	[6,41]
Psychotropic medication	Adjunct for OCD, anxiety, mood lability, insomnia, or severe behavioural symptoms when clinically indicated.	Consensus guidance; extrapolated from pediatric psychiatry rather than PANS-specific trials.	Start low and titrate cautiously; activation, agitation, and adverse effects can be difficult to distinguish from flares.	[6,41]
Targeted antimicrobial treatment	Treat documented or strongly suspected GAS or other bacterial infection using standard pediatric practice.	Consensus infection guidance; observational and survey literature.	Avoid attributing all flares to infection; culture/serology should be interpreted in clinical context.	[4,5,6,39]
Antimicrobial prophylaxis	Consider only in selected recurrent GAS-associated cases after specialist review.	Consensus guidance and limited observational evidence.	Not routine for all PANS; stewardship, resistance, gastrointestinal effects, allergy risk, and microbiome disruption must be weighed.	[4,5,39]
NSAIDs or short corticosteroid courses	May be considered for suspected inflammatory flares after infection and safety concerns have been addressed.	Uncontrolled clinical experience, consensus guidance, and systematic reviews.	Evidence is low certainty; monitor renal, gastrointestinal, metabolic, mood, sleep, and immunosuppressive risks.	[5,42]
IVIG	Possible option for selected moderate-to-severe, relapsing, or refractory immune-mediated presentations.	Mixed trial and observational literature, including severe-cohort clinical experience, summarized in systematic reviews and consensus guidance.	Expensive; adverse effects include headache, aseptic meningitis, thrombosis risk, hemolysis, and infusion reactions; not a diagnostic test.	[4,5,42,43,45]
Therapeutic plasma exchange	Reserved for severe, disabling, or refractory cases after specialist evaluation.	Randomized trial and uncontrolled cohort literature, plus consensus guidance.	Invasive; requires vascular access and expertise; risks include line complications, hypotension, bleeding, infection, and relapse.	[42,43,44]
Tonsillectomy/adenoidectomy	Not recommended solely as disease-modifying PANS therapy; use standard ENT indications.	Systematic reviews find insufficient evidence for PANS/PANDAS symptom control.	Avoid surgery unless conventional indications exist.	[4,5]
Microbiome-directed strategies	Investigational adjunctive concept, not established treatment.	Narrative mechanistic review and preclinical/associative data.	No controlled PANS-specific efficacy data; avoid replacing evidence-based psychiatric or medical care.	[36,37]

Abbreviations: CBT, cognitive-behavioural therapy; ENT, ear, nose, and throat; ERP, exposure and response prevention; GAS, group A *Streptococcus*; IVIG, intravenous immunoglobulin; NSAID/NSAIDs, non-steroidal anti-inflammatory drug(s); OCD, obsessive–compulsive disorder; PANDAS, Pediatric Autoimmune Neuropsychiatric Disorders Associated with Streptococcal infections; PANS, pediatric acute-onset neuropsychiatric syndrome. Note: The table is a clinical evidence summary, not a treatment protocol.

**Table 3:** Practical evaluation framework for suspected infection-associated PANS/PANDAS.

Evaluation Step	What it Clarifies	Practical Elements	Clinical Interpretation
Confirm acute syndromic pattern	Whether the presentation fits a PANS/PANDAS-type onset rather than a gradual psychiatric trajectory.	Identify the last period at baseline; document abrupt OCD or severe food restriction; record accompanying domains such as anxiety, tics, sleep disruption, urinary symptoms, cognitive decline, regression, or rage.	A clear temporal inflection supports PANS evaluation, but criteria still require exclusion of better established neurological, medical, or psychiatric explanations [2,10].
Assess immediate safety and medical stability	Whether urgent intervention is needed before etiologic attribution.	Assess hydration, nutrition, weight change, food and fluid refusal, suicidality, aggression, psychosis, catatonia, severe insomnia, medication exposure, and caregiver capacity.	Medical instability or psychiatric danger should trigger urgent care regardless of whether infection-associated PANS is later confirmed [2,6].
Use targeted infectious assessment	Whether there is a treatable infection or plausible post-infectious timeline.	Consider GAS testing when pharyngitis, exposure, scarlet-fever-like features, or recurrent streptococcal flares are present. Test for *Mycoplasma pneumoniae*, EBV, SARS-CoV-2, influenza, or Lyme disease only when history, examination, epidemiology, and local guidance support it.	Positive tests require clinical context because carriage, past exposure, non-specific serology, and coincidental infection can mislead management [2,6,39].
Screen for mimics and comorbidities	Whether the same symptoms are better explained by another disorder or by overlapping conditions.	Look for autoimmune encephalitis red flags, Sydenham chorea, seizures, delirium, endocrine disease, medication or substance effects, primary OCD, Tourette syndrome, eating disorders, sleep disorders, and systemic autoimmune disease.	Differential diagnosis is central to safe care; red flags should shift the pathway toward neurology, infectious diseases, rheumatology, emergency medicine, or psychiatry as appropriate [2,6,38].
Measure baseline severity and function	How to track course, response, relapse, and adverse effects.	Record OCD time burden, food intake, weight, school attendance, sleep duration, urinary symptoms, rage episodes, tics, anxiety, handwriting or academic decline, and family accommodation.	Repeated measures reduce reliance on global impressions during fluctuating illness and help separate treatment response from natural waxing and waning [6,41].
Create a shared multidisciplinary plan	How to align psychiatric, pediatric, infectious, and immunological decision-making.	Explain diagnostic uncertainty; validate abrupt deterioration; clarify that psychiatric care does not exclude immune contribution; document treatment indications, duration, outcome targets, adverse-event monitoring, and stopping rules.	Shared planning reduces polarized care, unnecessary escalation, and therapeutic delay while keeping higher-risk interventions proportional to severity and diagnostic confidence [6,40,42].

Abbreviations: EBV, Epstein–Barr virus; GAS, group A *Streptococcus*; OCD, obsessive–compulsive disorder; PANDAS, Pediatric Autoimmune Neuropsychiatric Disorders Associated with Streptococcal infections; PANS, pediatric acute-onset neuropsychiatric syndrome; SARS-CoV-2, severe acute respiratory syndrome coronavirus 2. Note: The framework is a practical synthesis of cited guidance and reviewed literature, not a formal diagnostic algorithm.

**Table 4:** Conceptual bridge between proposed mechanisms and clinical interpretation in infection-associated PANS/PANDAS.

Mechanistic Domain	Core Concept	Potential Clinical Expression	Interpretive Implication
Molecular mimicry and antineuronal antibodies	Post-infectious antibodies may cross-react with neuronal targets, particularly in basal ganglia-related circuits.	Abrupt OCD symptoms, tics, emotional lability, motor changes, or relapse after a compatible infection.	Biologically plausible, especially for GAS-associated PANDAS, but antibody findings remain insufficient as stand-alone diagnostic markers [19,22,25].
D1R/D2R and CaMKII signaling	Dopamine receptor autoantibodies and antibody-mediated neuronal signaling may influence neurotransmitter pathways.	Changes in compulsivity, habit learning, tic expression, threat response, or emotional regulation.	Anti-D1R/anti-D2R findings are mechanistically important, but clinically validated thresholds and treatment-predictive cutoffs are lacking [20,21,23,24].
Blood–brain barrier vulnerability and cytokine effects	Systemic inflammation may alter barrier permeability and expose neural circuits to peripheral immune mediators.	Sleep disruption, appetite change, pain sensitivity, fatigue, cognitive inefficiency, autonomic or urinary symptoms, and heightened anxiety.	Inflammation may amplify symptoms without proving a specific pathogen as the cause; timing and objective infection data remain essential [29,30].
Microglial, cholinergic interneuron, and CSTC circuit amplification	Immune activation may affect circuits governing motor inhibition, threat learning, habit formation, and behavioral flexibility.	Rage episodes, regression, handwriting deterioration, motor symptoms, avoidance, reassurance seeking, or sudden functional decline.	Circuit-level models help explain symptom clustering across triggers, but they do not eliminate the need to evaluate psychiatric, neurological, and medical mimics [31,32,33,34].
Gut–oral–brain and microbiome–immune interactions	Microbiome patterns, mucosal immune tone, antibiotic exposure, diet, LPS/endotoxemia, and inflammatory metabolites may influence host susceptibility.	Gastrointestinal symptoms, dietary restriction, fluctuating inflammation, or symptom changes after infection or antibiotic exposure.	The concept is hypothesis-generating; microbiome-directed approaches should remain adjunctive and should not replace established care [35,36,37].
Pluralistic treatment proportionality	Different children may have overlapping infectious, inflammatory, psychiatric, developmental, and environmental contributors.	Mixed or fluctuating responses to CBT/ERP, antibiotics, anti-inflammatory agents, IVIG, or plasma exchange.	Management should match treatment intensity to severity, diagnostic confidence, differential diagnosis, and adverse-effect burden rather than presume one universal pathway [4,5,40,42].

Abbreviations: CaMKII, calcium/calmodulin-dependent protein kinase II; CBT/ERP, cognitive-behavioural therapy with exposure and response prevention; CSTC, cortico–striato–thalamo–cortical; D1R, dopamine receptor D1; D2R, dopamine receptor D2; GAS, group A *Streptococcus*; IVIG, intravenous immunoglobulin; LPS, lipopolysaccharide; OCD, obsessive–compulsive disorder; PANDAS, Pediatric Autoimmune Neuropsychiatric Disorders Associated with Streptococcal infections; PANS, pediatric acute-onset neuropsychiatric syndrome. Note: The table summarizes proposed mechanisms and does not assign causality to any individual case.

## Data Availability

No new data were created or analysed in this study. Data sharing is not applicable to this article.

## Abbreviations

AAP	American Academy of Pediatrics
BBB	Blood–brain barrier
CaMKII	Calcium/calmodulin-dependent protein kinase II
CBT	Cognitive-behavioural therapy/cognitive-behavioral therapy
CIN	Cholinergic interneuron
CNS	Central nervous system
COVID-19	Coronavirus disease 2019
CSF	Cerebrospinal fluid
CSTC	Cortico–striato–thalamo–cortical
D1R	Dopamine D1 receptor
D2R	Dopamine D2 receptor
DNase B	Deoxyribonuclease B; used in the manuscript as anti-DNase B titers
EBV	Epstein–Barr virus
EEG	Electroencephalography
ERP	Exposure and response prevention
ENT	Ear, nose, and throat
FRAA/FRAAs	Folate receptor alpha autoantibody/autoantibodies
GAS	Group A *Streptococcus*
GRADE	Grading of Recommendations Assessment, Development and Evaluation
IVIG	Intravenous immunoglobulin
LPS	Lipopolysaccharide
MEDLINE	Medical Literature Analysis and Retrieval System Online
MRI	Magnetic resonance imaging
NCT	National Clinical Trial identifier used by ClinicalTrials.gov
NSAID/NSAIDs	Non-steroidal anti-inflammatory drug(s)
OCD	Obsessive–compulsive disorder
PANDAS	Pediatric Autoimmune Neuropsychiatric Disorders Associated with Streptococcal infections
PANS	Pediatric acute-onset neuropsychiatric syndrome
PRISMA-ScR	Preferred Reporting Items for Systematic reviews and Meta-Analyses extension for Scoping Reviews
PET	Positron emission tomography
PTEC	PANS/PANDAS and Related Inflammatory Brain Disorders Treatment Evaluation Checklist
SANRA	Scale for the Assessment of Narrative Review Articles
SARS-CoV-2	Severe acute respiratory syndrome coronavirus 2
TSPO	Translocator protein
